# Click Modification for Polysaccharides via Novel Tunnel Transmission Phenomenon in Ionic Liquids

**DOI:** 10.34133/2022/9853529

**Published:** 2022-02-10

**Authors:** Yan Zhou, Jinming Zhang, Yaohui Cheng, Xin Zhang, Jin Wu, Jun Zhang

**Affiliations:** ^1^CAS Key Laboratory of Engineering Plastics, CAS Research/Education Center for Excellence in Molecular Sciences, Institute of Chemistry, Chinese Academy of Sciences (CAS), Beijing 100190, China; ^2^University of Chinese Academy of Sciences, Beijing 100049, China

## Abstract

It is extremely difficult to achieve a rapid and efficient modification of natural polysaccharides, due to the intrinsic strong hydrogen bonding networks and the slow mass transfer process during the reaction process. Herein, we found a fascinating anion-tunnel transmission phenomenon in the imidazolium-based ionic liquids with carboxylate anions. A novel click esterification of natural polysaccharides thus was demonstrated under a catalyst-free condition within a very short reaction time of 15 min at 0-80°C. Such a super-rapid and highly efficient modification strategy is available for various polysaccharides (cellulose, starch, inulin, pullulan, dextran, and xylan), different esterification reactions (acetification, propionation, benzoylation, and cyclohexyl formylation), and high concentrations, claiming a revolutionary potential in polysaccharide chemistry industries.

## 1. Introduction

As the natural renewable resource, polysaccharides are polymeric carbohydrate structures, in which monosaccharide (e.g., glucose, fructose, and galactose) or disaccharide (e.g., sucrose and lactose) units are covalently joined by O-glycosidic bonds in either a linear or a branched configuration [1–4]. Polysaccharides serve as stores of energy, as in starch and glycogen (branched polysaccharide of glucose), and as a structural component of cell walls, as in cellulose and chitin (linear polysaccharide of glucose). They have many attractive advantages, such as tremendous reserves, natural renewability, complete biodegradability, and outstanding biocompatibility and hence are considered as ecofriendly raw materials to promote the sustainability of human society. More interestingly, there are numerous reactive hydroxyl groups along polysaccharides chains; thus, it is easily available to chemical modifications of polysaccharides, which can significantly improve the solubility, processability, strength, flexibility, stability, and other physical properties of polysaccharide materials, meanwhile endowing them with new functional properties [5–11]. However, massive hydroxyl groups spontaneously form strong hydrogen bond networks, so it is extremely difficult to dissolve and modify polysaccharides. For example, the famous and commonly used cellulose acetate, including cellulose diacetate (CDA) and cellulose triacetate (CTA), can only be produced through the heterogeneous chemical modification in industry [12]. The heterogeneous reactions lead to several undesirable problems, such as complex synthesis process, nonuniform distribution of substituents, considerable degradation of cellulose, and long reaction time. Moreover, it is impossible to directly control the chemical structure of the resultant cellulose acetate. Therefore, it is appealing to replace the traditional heterogeneous process with the homogeneous process by developing effective solvents for polysaccharides [5, 11, 13–15]. But, the previous reported homogeneous derivatization of polysaccharides is usually inefficient, because the high viscosity of polysaccharide solutions results in a slow mass transfer process, which originates from the intrinsic high molecular weight of polysaccharides. For example, in the most commonly used solvents of cellulose, N,N′-dimethylacetamide/lithium chloride (DMAc/LiCl) and ionic liquids (ILs), the acetylation and benzoylation usually take a long-term period of more than 2 h to reach equilibrium [16–18]. The successful propionation and butyrylation of cellulose need much more time and harsh reaction conditions [19, 20]. In the current systems, the transmission of acylation reagents is mainly driven by diffusion, so the high viscosity will lead to a sluggish reaction speed. Moreover, the slow mass transmission tends to cause the local inhomogeneity in the reaction system, so the intensified stirring force and/or extended reaction time are always necessary to eliminate the local inhomogeneity phenomenon. But these operations are likely to induce the depolymerization of polysaccharides. Therefore, it is challenging and practical to realize a fast, efficient, and homogeneous modification of polysaccharides, after overcoming their intrinsic strong hydrogen bonding networks and a slow mass transfer process during the reaction process.

**Figure 1:**
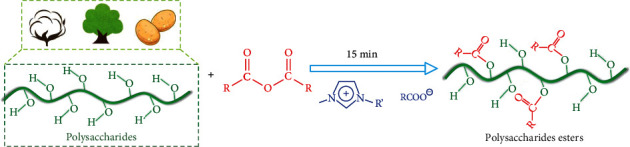
Click esterification of polysaccharides in imidazolium-based ILs with carboxylate anions.

In this work, we found an extremely rapid anion-tunnel transmission phenomenon in the imidazolium-based ILs with carboxylate anions (R′mimRCOO), based on the hydrogen bond network formed by imidazolium cations and carboxylate anions. Then, a general, easy-to-scale, and homogeneous esterification is proposed to achieve a click modification of polysaccharides (Figure 1).

## 2. Experimental Section

### 2.1. Synthesis of R′mimRCOO Ionic Liquid

Taking 1-butyl-3-methylimidazolium acetate (BmimAc), for example, the synthesis of BmimAc underwent a process of ion exchange and neutralization. Firstly, the 1-butyl-3-methylimidazolium chloride (BmimCl) ionic liquid aqueous solution was used to treat with a 717-type anion exchange resin; thus, the 1-butyl-3-methylimidazolium hydroxide (BmimOH) solution was acquired. Then, the BmimOH aqueous solution was neutralized with acetic acid. After a rotary evaporation to remove the water, the BmimAc ionic liquid was obtained.

### 2.2. Acylation of Cellulose in Ionic Liquids

Taking cellulose acetylation for example, the certain amount of acetic anhydride or acetyl chloride was added into the cellulose/R′mimAc solutions with vigorous mechanical stirring at a given temperature. After the required time, the resultant products were isolated as the water-insoluble or methanol-insoluble fraction, filtered and washed three times with deionized water or methanol. Then, they were redissolved in DMSO, precipitated again into water or methanol, and thoroughly washed with the same solvent. Finally, these products were dried under vacuum at 80°C. The DS of all products was analyzed by the NMR method.

## 3. Results and Discussion

### 3.1. Click Acetylation of Cellulose

**Figure 2:**
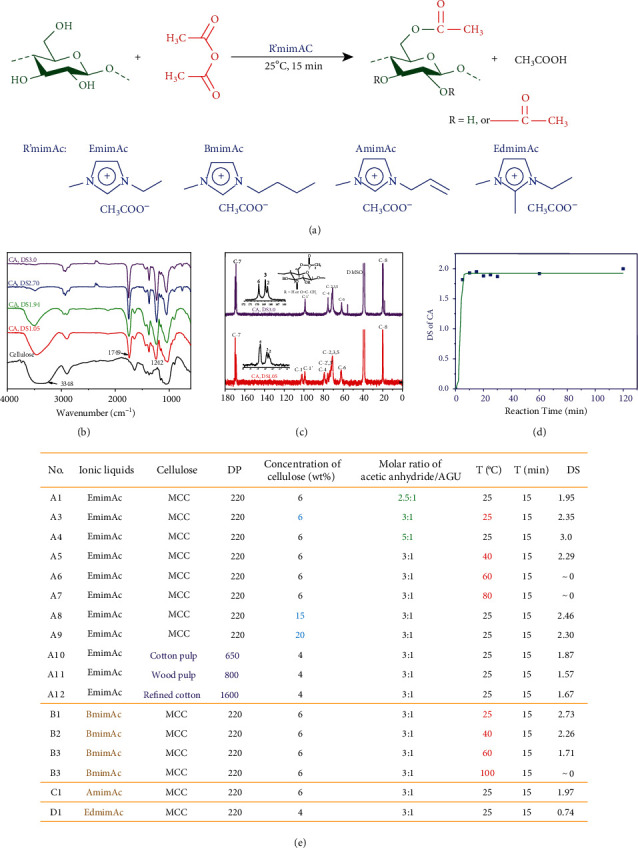
(a) Synthesis route of the click acetylation of cellulose. (b) FTIR spectra of CA. (c) ^13^C-NMR spectra of CA. (d) Effect of reaction time on the DS of CA synthesized in EmimAc with a molar ratio of 2.5 : 1 at room temperature. (e) Reaction conditions and results of the click acetylation of cellulose in RmimAc.

Taking the famous cellulose acetate (CA) for example, a homogeneous click acetylation of cellulose is accomplished at 25°C for 15 min in four kinds of imidazolium-based ILs with acetate anions (RmimAc), 1-ethyl-3-methylimidazolium acetate (EmimAc), BmimAc, 1-allyl-3-methylimidazolium acetate (AmimAc), and 1-ethyl-2,3-dimethylimidazolium acetate (EdmimAc) (Figure 2(a)). In FTIR spectra of acetylated cellulose products (Figure 2(b)), the strong absorption peaks at 1749 cm^−1^ (C=O stretching) and 1242 cm^−1^ ((O)C–O stretching) are observed, confirming the formation of the ester group. Moreover, as the degree of substitution (DS) increases, the intensity of the absorption band at 3200-3700 cm^−1^ (OH stretching) obviously decreases, even disappears (DS = 3.0), indicating that the hydroxyl groups in cellulose have been acylated. In addition, the ^13^C-NMR spectra display that the peak of methyl carbon is at 19 ppm, and the peak of carbonyl carbon is in the range of 168-171 ppm (Figure 2(c)). According to ^13^C-NMR spectra of CA (Figure 2(c)), the reaction activities of three hydroxyl groups at the 2, 3, and 6 positions can be calculated qualitatively [17]. Clearly, the acetylation reaction is preferred at 6-OH, and the order of reactivity is 6 − OH > 2 − OH ≈ 3 − OH. These FTIR and NMR results are in accordance with those previous reports [12, 17] and prove that the CA is successfully synthesized.

The acetylation of cellulose is extremely rapid and highly efficient in RmimAc. It is clear that, at a relatively low temperature, such as 25°C and 40°C, only 15 min is needed to reach the equilibrium state (Figures 2(d), and 2(e) and Table S1). Further increasing the reaction time has a negligible effect on the DS of the products. The reaction rate is much higher than those of cellulose acylation in 1-allyl-3-methylimidazolium chloride (AmimCl), BmimCl, and DMAc/LiCl reported in the literatures. For example, in BmimAc, the DS of CA can reach 2.73 after 15 min at 25°C when a molar ratio of acetic anhydride and anhydroglucose unit (AGU) is 3 : 1. By contrast, in AmimCl, the DS of CA reaches 2.74 after 23 h at 80°C with a molar ratio of 5 : 1 [17], and in BmimCl, the DS of CA reaches 2.72 after 2 h at 80°C with a molar ratio of 5 : 1 [18]. In addition, the highest conversion efficiency of acylating reagents achieves 90%, which is much higher than those of the acylating reagents in the previous reports [16–18]. Moreover, the click acetylation is available for various celluloses with different degrees of polymerization (DP) and sources, including microcrystalline cellulose (MCC), wood pulp, cotton pulp, and refined cotton, and for high cellulose concentrations, even if a 20 wt% cellulose/IL solution was used. Therefore, it can be concluded that RmimAc ILs are the optimum media for the homogeneous acetylation of cellulose.

It is generally accepted that an increase in reaction temperature will speed up the acylation [21, 22]; however, this click acetylation is just the opposite (Figure 2(e)). Applying a molar ratio of 3 : 1 and a reaction time of 15 min, a DS of 2.73 in CA is achieved in BmimAc at 25°C, whereas, surprisingly, the DS decreases to 2.26 with the increasing temperature to 40°C and 1.71 at 60°C. The same phenomenon was observed in EmimAc. When the temperature is above 60°C, the acetylated cellulose products obtained after 15 min are not soluble in powerful solvents of CDA (such as DMSO, DMAc, and DMF), indicating that the reaction extent between cellulose and acetic anhydride is insignificant and the DS of products is nearly close to zero. Such a general click acetylation with two fascinating phenomena, including an inverse correlation between the reaction rate and the reaction temperature and a perfect uncorrelation between the reaction rate and the cellulose concentration, demonstrates a revolutionary potential in cellulose chemistry industry.

### 3.2. Mechanism of Click Acetylation of Cellulose

**Figure 3:**
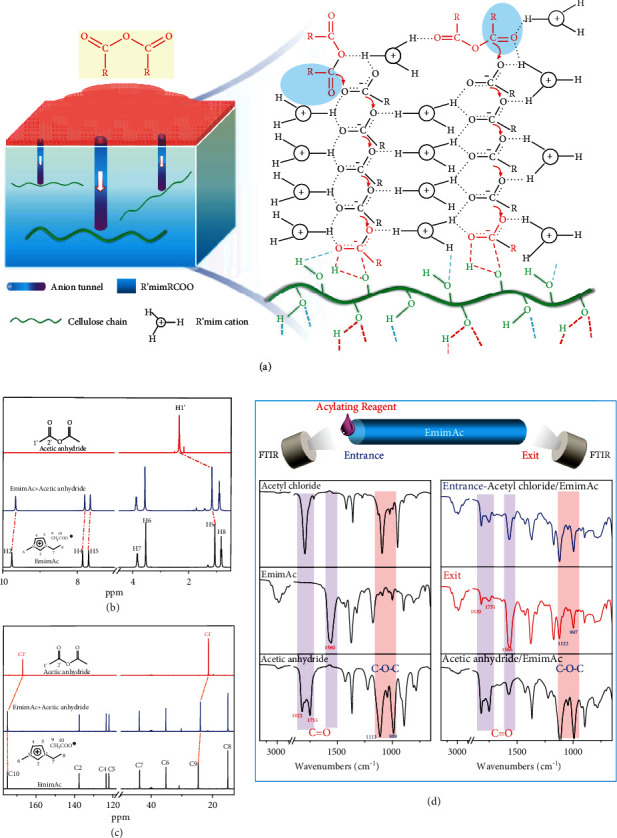
(a) Schematic diagram of the transmission of acylating reagents in R′mimRCOO. (b) ^1^H-NMR spectra of acetic anhydride, EmimAc, and the mixture of acetic anhydride and EmimAc. (c) ^13^C-NMR spectra of acetic anhydride, EmimAc, and the mixture of acetic anhydride and EmimAc. (d) Comparison of FTIR spectra of EmimAc, acetyl chloride, acetic anhydride, acetic anhydride/EmimAc, entrance, and exit.

Such a rapid and fascinating acetylation of cellulose in R′mimAc is impressive and appealing, which inspires us to explore its mechanism. During the acetylation process, the used acylating reagents, acetic anhydride and acetyl chloride, are highly active. The reaction between acylating reagents and hydroxyl groups is very quick, so the rate-determining step is the mass transfer process of acylating reagents. However, it is well-known that the mass transfer process is very slow in polymer solutions by diffusion, due to the high viscosity. Thus, it seems that the transmission of acylating reagents in R′mimAc is not a conventional diffusion process. Previous studies claim that the anions and cations in ILs would like to form an interionic hydrogen-bonding network structure, in which the anions and cations arrange alternately at different rows (Figure 3(a)) [23–26]. We speculate that the anion channel in R′mimAc provides a quick tunnel transmission effect on the mass transfer of acetylating reagents (Figure 3(a) and S1), which is similar to the famous H^+^ and OH^−^ hopping processes via the hydrogen bonding network in water [27]. As a result, a super-rapid acetylation of cellulose in R′mimAc is accomplished successfully.

Specifically, the acetylating reagents added into the cellulose/R′mimAc solution interact with the anions and cations in the first layer of R′mimAc by the hydrogen bonding interactions (Figure 3(a) and S1). The oxygens of acetate anions interact with the electron-deficient acyl carbons of the acetylating reagents, and the active hydrogens of [R′mim]^+^ cations interact with the electron-rich oxygens or chlorines in the acetylating reagents. Because of the dynamic reversibility of the hydrogen bonding interactions, the acetylating reagents are transferred into the first layer. Subsequently, the transmission process is repeated, and the acetylating reagents are transferred into the second layer. After a layer-by-layer transmission in the acetate anion channel, the acetylating reagents are rapidly transmitted into the interior of the cellulose/R′mimAc solution. Therefore, the rapid acetylation of cellulose is realized.

Experiments were designed to prove the above mechanism. Once acetic anhydride is added into EmimAc, the peak of methyl proton in acetic anhydride at 2.33 ppm and the peak of methyl proton in acetate anion at 1.07 ppm merge to a new peak at 1.17 ppm in ^1^H-NMR spectra (Figure 3(b)). In ^13^C-NMR spectra (Figure 3(c)), the peak of acyl carbon in acetic anhydride at 167.05 ppm and the peak of acyl carbon in acetate anion at 174.93 ppm merge to a new peak at 174.90 ppm, and the peak of methyl carbon in acetic anhydride at 21.33 ppm and the peak of methyl carbon in acetate anion at 24.65 ppm merge to a new peak at 23.96 ppm. These phenomena demonstrate that the acetic anhydride and the acetate anions in EmimAc become one by the dynamic hydrogen bonding interactions.

FTIR spectra confirm the quick transmission of acylating reagents in R′mimAc directly (Figure 3(d) and S2). Two drops of acetic anhydride or acetyl chloride were added on the left of a slender glass tube with EmimAc. After 2 min, the FTIR spectra of the liquids at the entrance (the left of the tube) and exit (the right of the tube) were recorded. At the entrance, there are three peaks belonging to carbonyl groups at 1820, 1751, and 1572 cm^−1^, and the C-O-C stretching vibration peaks at 1120 and 995 cm^−1^, indicating that acetyl chloride is immediately changed into acetic anhydride once acetyl chloride is dropped into EmimAc. More interestingly, at the exit end, there are also three carbonyl peaks at 1820, 1751, and 1566 cm^−1^, and the C-O-C stretching vibration peaks at 1122 and 997 cm^−1^. These results confirm that the acetylating reagents, either acetic anhydride or acetyl chloride, have been transferred from the entrance to the exit in a very short time. Subsequently, a visual experiment has been done to confirm the quick transmission of acylating reagents in R′mimAc. The acetyl chloride with a small amount of oil red O was added into the EmimAc (94.2 cP at 298 K). After only 2 min, the red liquid reaches the bottom of the EmimAc (Figure S3). By contrast, in AmimCl (2086.1 cP at 298 K) and 1-hydroxyethyl-3-methylimidazolium bis(trifluoromethylsulfonyl)imide (HemimTf_2_N, 84.4 cP at 298 K), the acetyl chloride with oil red O gives an extremely slow transmission rate (Figures S4 and S5). Thus, the actual transmission rate of acetylating reagents in R′mimAc is super-rapid by the anion tunnel transmission effect.

Since the acetylating reagents exhibit a rapid transmission by using the anion channel in R′mimAc, 1-ethyl-3-methylimidazolium chloride (EmimCl) was added into EmimAc to disrupt the original hydrogen bonding network of EmimAc and form the new network containing [Emim]^+^ cations, Ac^−^ anions, and Cl^−^ anions, in which the rapid transmission of the acetylating reagents will be inhibited (Figure S6). As a result, the reaction speed of the acetylation of cellulose with acetic anhydride will slow down. Actually, the experimental results prove our conjecture. As the content of EmimCl in the EmimCl/EmimAc mixture increases, the DS of CA decreases under the same reaction conditions (Table S2). When the content of EmimCl is above 30%, only a small part of acetylated cellulose products can be dissolved in DMSO. Moreover, as the content of EmimCl increases, the amount of the soluble part in the products decreases dramatically. In the EmimAc/AmimCl mixture, the same phenomenon has been observed (Figure S7).

Likewise, as the reaction temperature increases, the rapid transmission of the acetylating reagents will be blocked also, because the high temperature will break the hydrogen bonding network in R′mimAc. Thus, the abnormal effect of the elevation of temperature on the reaction rate (Figure 2(e)) confirms that the rapid tunnel transmission in R′mimAc is essential to achieve a rapid and efficient acetylation.

In summary, the fascinating anion-tunnel transmission effect via dynamic hydrogen bonding interactions in ionic liquids R′mimRCOO is responsible for the rapid and highly efficient homogeneous acylation of cellulose. This mechanism can explain several experimental phenomena. For instance, EdmimAc is the least effective solvent for homogeneous acetylation of cellulose in four acetate-based ILs. That is because the substitution of 2-H in the cation by the methyl group changes the hydrogen bonding network in ILs, which is unfavorable to the rapid tunnel transmission of the acetylating reagents. For another example, when the acylation of cellulose with propionic anhydride or benzoyl chloride is carried out in RmimAc, the cellulose mixed esters, cellulose acetate propionate, or cellulose acetate benzoate are obtained. Moreover, the content of acetyl groups is 2-3 times more than that of another acylation group in the final products.

### 3.3. Various Click Esterification of Polysaccharides

**Figure 4:**
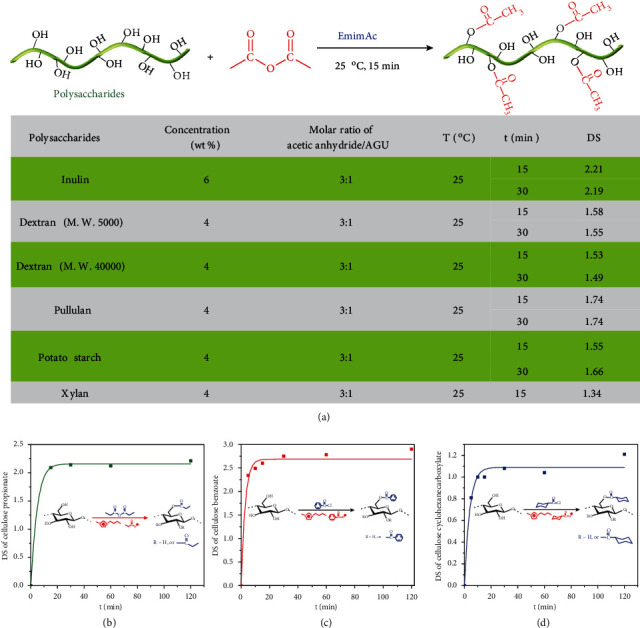
(a) Rapid acetylation of polysaccharides at 25°C. (b) Effect of reaction time on the DS of cellulose propionate synthesized in 1-butyl-3-methylimidazolium propionate ([Bmim][CH_3_CH_2_COO]) with a molar ratio of 3 : 1 at 80°C. (c) Effect of reaction time on the DS of cellulose benzoate synthesized in 1-butyl-3-methylimidazolium benzoate ([Bmim][PhCOO]) with a molar ratio of 3 : 1 at 80°C. (d) Effect of reaction time on the DS of cellulose cyclohexanecarboxylate synthesized in 1-butyl-3-methylimidazolium cyclohexanecarboxylate ([Bmim][ChCOO]) with a molar ratio of 3 : 1 at 80°C.

The above novel click reaction principle is suitable to various polysaccharides, including cellulose, starch, inulin, pullulan, dextran, and xylan and to multifarious acylation reactions, such as acetylation, propionation, benzoylation, and cyclohexyl formylation. In EmimAc, different kinds of polysaccharides with different molecular weight (M.W.) values, such as potato starch, inulin, pullulan, dextran with a M.W. of 5000, dextran with a M.W. of 40000, and xylan, give a rapid acetylation under catalyst-free condition within 15 min at 25°C (Figure 4(a)). In addition, via using R′mimRCOO ILs with the corresponding carboxylate anions as the reaction media, multifarious acylation reactions, such as propionation, difficult cyclohexyl formylation, and benzoylation, can be accomplished under a catalyst-free condition within 15 min also (Figures 4(b)–4(d)). Polysaccharide esters with controllable DS values were readily obtained by adjusting the molar ratio of acylating reagents and the reaction temperature (Tables S3-S5).

## 4. Conclusions

Taking advantage of the characteristic network structure in ionic liquids, a tunnel transmission effect for acylation reagents in the imidazolium-based ionic liquids with carboxylate anions was found via dynamic hydrogen bonding interactions. Based on this novel phenomenon, a click modification of various natural polysaccharides was demonstrated. The click esterification reactions were effectively accomplished under mild and catalyst-free conditions within a very short reaction time of 15 min, which is much faster and more efficient than the previous reports. More interestingly, the reaction rates were negligibly dependent on the concentration and molecular weight of polysaccharides. This novel click reaction principle was suitable to different polysaccharides, including cellulose, starch, inulin, pullulan, dextran, and xylan, and to multifarious acylation reactions, such as acetylation, propionation, benzoylation, and cyclohexyl formylation. Such a rapid, efficient, general, and easy-to-scale reaction with an excellent adaptation to the viscous solutions is advanced and revolutionary to the current polysaccharide chemistry process. More interestingly, we predict that, according to this tunnel transmission principle, many chemical processes can be accomplished rapidly and efficiently by using the appropriate ILs as the reaction media, such as the synthesis of protein medicines.

## Data Availability

All data required to support the conclusions are presented in the main text and the supplementary materials.

## Supplementary Materials

Supplementary Materials Experimental section; polysaccharide acylation under different conditions; mechanism of rapid transmission; FTIR spectra; photographs of the mass transfer process.
