# Supplementary material for: Click Modification for Polysaccharides via Novel Tunnel Transmission Phenomenon in Ionic Liquids
Source: Research (Wash D C). 2022 Feb 10;2022:9853529. doi: 10.34133/2022/9853529 (PMC8857704; doi:10.34133/2022/9853529)
Supplement: Supplementary Materials — Experimental section; polysaccharide acylation under different conditions; mechanism of rapid transmission; FTIR spectra; photographs of the mass transfer process. [file 9853529.f1.doc]

**Supporting Information**

Click Modification for Polysaccharides via Novel Tunnel Transmission Phenomenon in Ionic Liquids

Yan Zhou^1,2^, Jinming Zhang^1^,*, Yaohui Cheng^1,2^, Xin Zhang^1,2^, Jin Wu^1^, Jun Zhang^1,2^,*

^1^ CAS Key Laboratory of Engineering Plastics, CAS Research/Education Center for Excellence in Molecular Sciences, Institute of Chemistry, Chinese Academy of Sciences (CAS), Beijing 100190, China

^2^ University of Chinese Academy of Sciences, Beijing 100049, China

**Supporting information content**

Number of pages: 8

Number of figures: 7

Number of tables: 5

**Experimental Section**

***Materials***

Microcrystalline cellulose was purchased from Beijing Fengli Jingqiu Pharmaceutical Co., Ltd. Cotton pulp was provided by Baoding Swan Fiber Co., Ltd. Wood pulp was obtained from Sichuan Pushi Acetati Co., Ltd. Refined cotton was supplied by Hubei Jinhanjiang Refined Cotton Co., Ltd. 1-Ethyl-3-methylimidazolium chloride (EmimCl, 99%), 1-butyl-3-methylimidazolium chloride (BmimCl, 99%), 1-ethyl-2,3-dimethylimidazolium chloride (EdmimCl, 99%) and 1-hydroxyethyl-3-methylimidazolium bis(trifluoromethylsulfonyl)imide (HemimTf_2_N, 99%) were acquired from Lanzhou Institute of Chemical Physics. 1-Allyl-3-methylimidazolium chloride (AmimCl, 99%) was synthesized in our lab.^1^ 717-Type anion exchange resin, acetic acid, propanoic acid, benzoic acid, cyclohexanecarboxylic acid, acetic anhydride and acetyl chloride were purchased from Sinopharm. Potato starch, inulin, pullulan, dextran with a M.W. of 5000, dextran with a M.W. of 40000 and xylan were bought from Sigma-Aldrich. Propionic anhydride, benzoyl chloride, cyclohexane carbonyl chloride and oil red O were obtained from TCI. Other reagents were used directly as received.

***Measurement***

FTIR spectra were recorded on FT-IR System 2000 spectrometer with the KBr technique. ^1^H-NMR spectra were acquired on a Bruker AV-400 NMR spectrometer with 16 scans at room temperature in DMSO-d_6_. A 20-μL aliquot of CF_3_COOH-d_1_ was added to shift the signals of the free hydrogens downfield. ^13^C-NMR spectra were measured with a Bruker AV 400 spectrometer with 4000-10000 scans at room temperature in DMSO-d_6_.

**Table S1.** Reaction conditions and results of the click acetylation of cellulose in BmimAc.


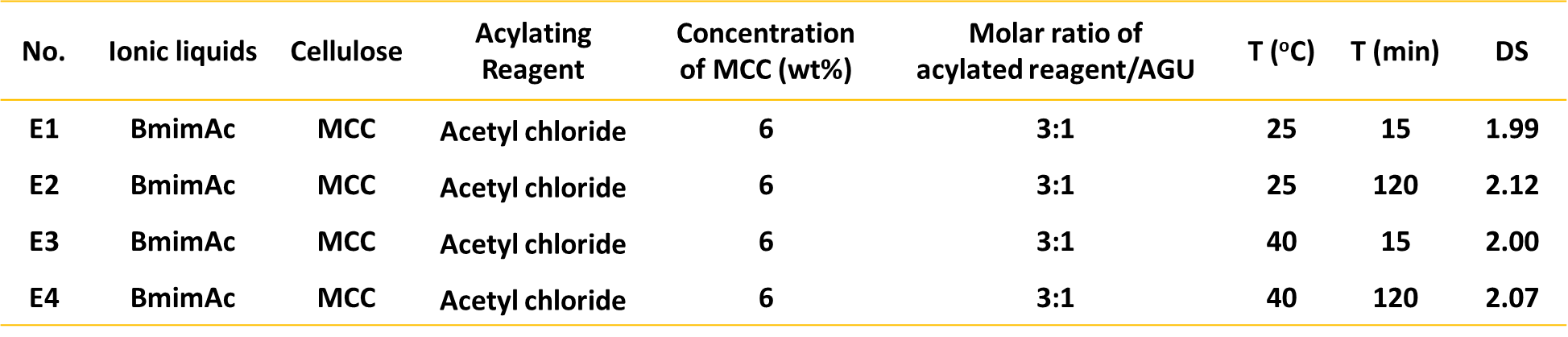


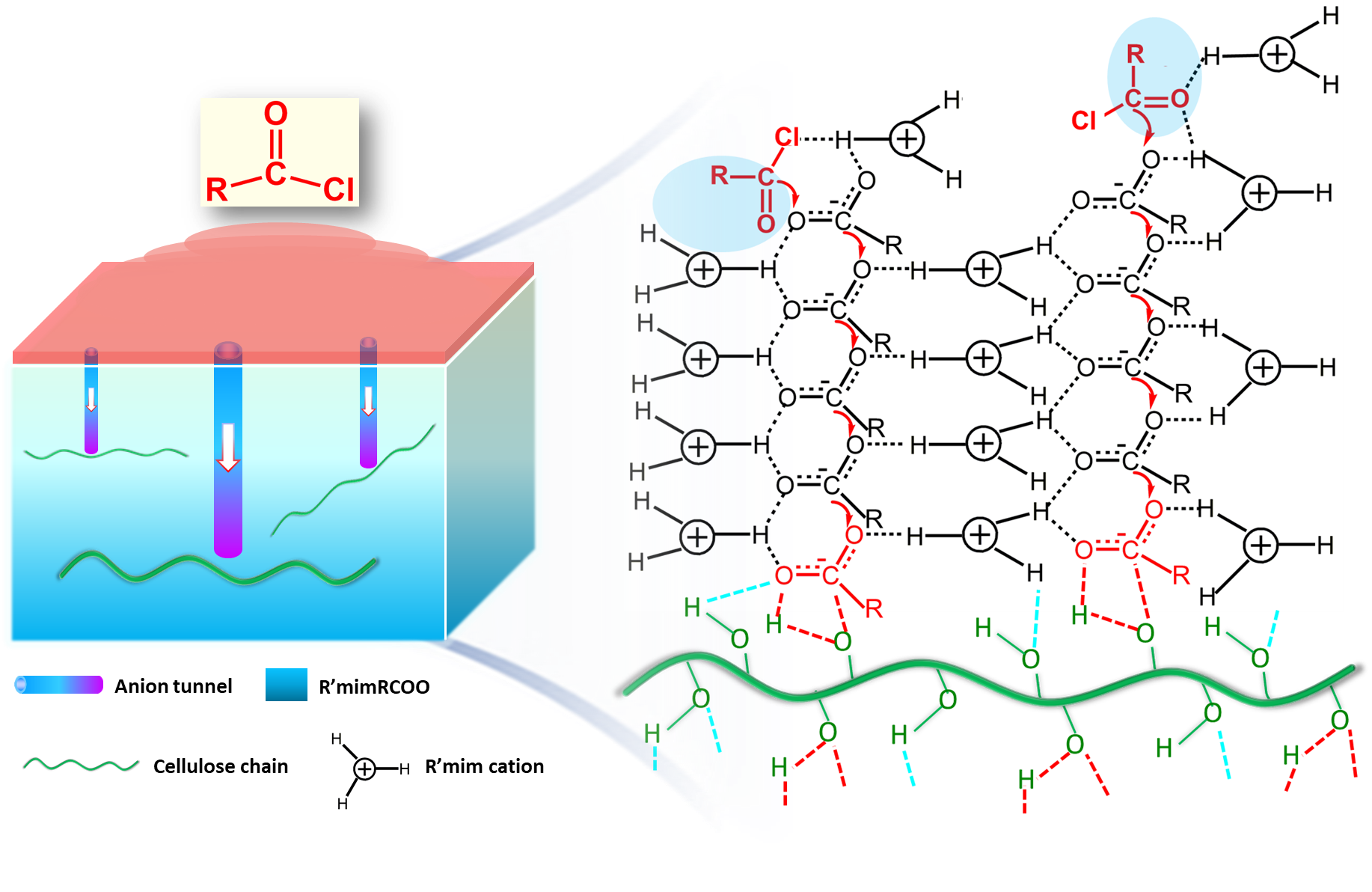


**Fig. S1.** Schematic diagram of the tunnel transmission of acyl chloride in RʹmimRCOO.


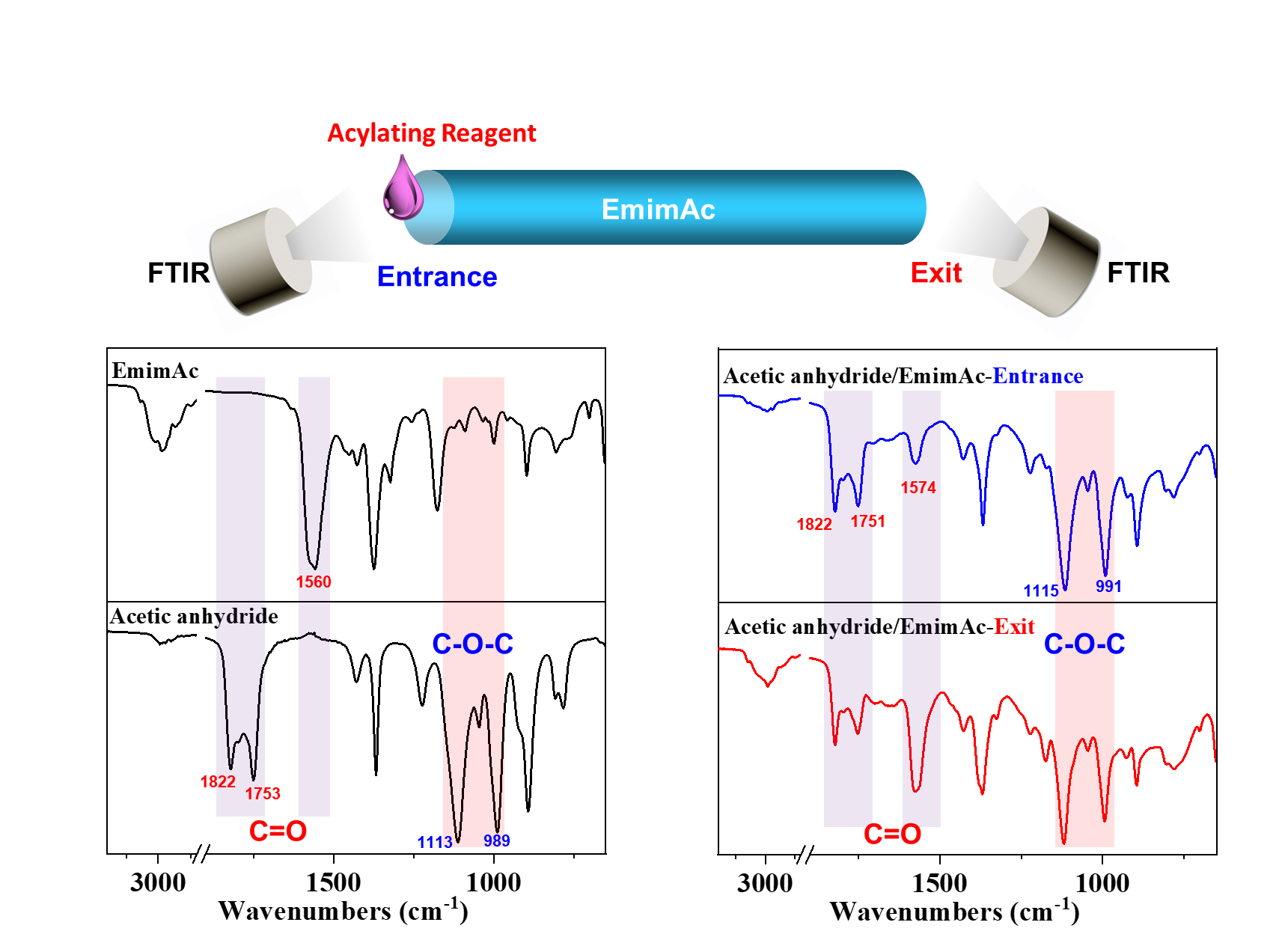


**Fig. S2.** Comparison of FTIR spectra of EmimAc, acetic anhydride, entrance and exit.


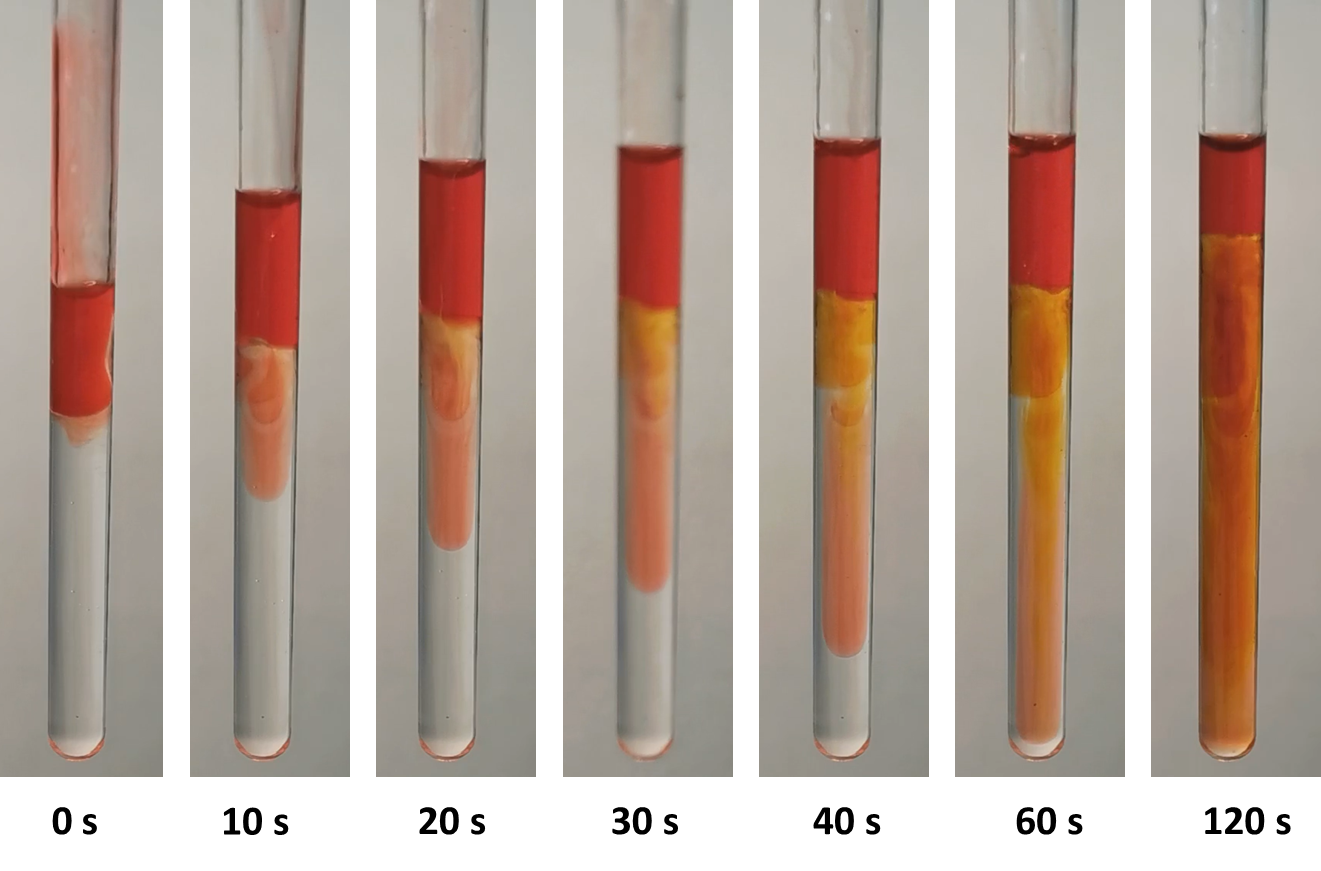


**Fig. S3.** Photographs of the addition of acetyl chloride with a small amount of oil red O into EmimAc.


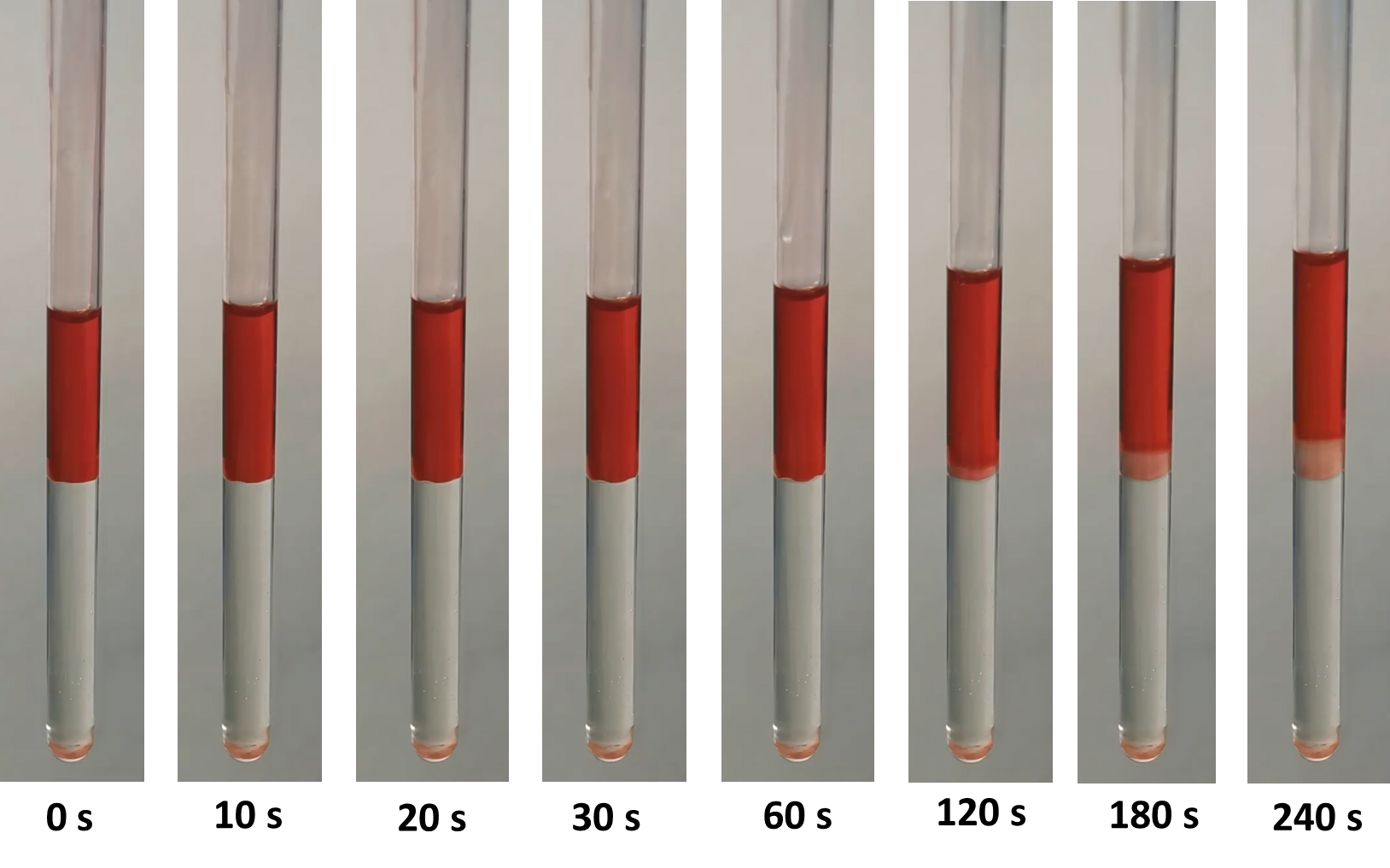


**Fig. S4.** Photographs of the addition of acetyl chloride with a small amount of oil red O into AmimCl.


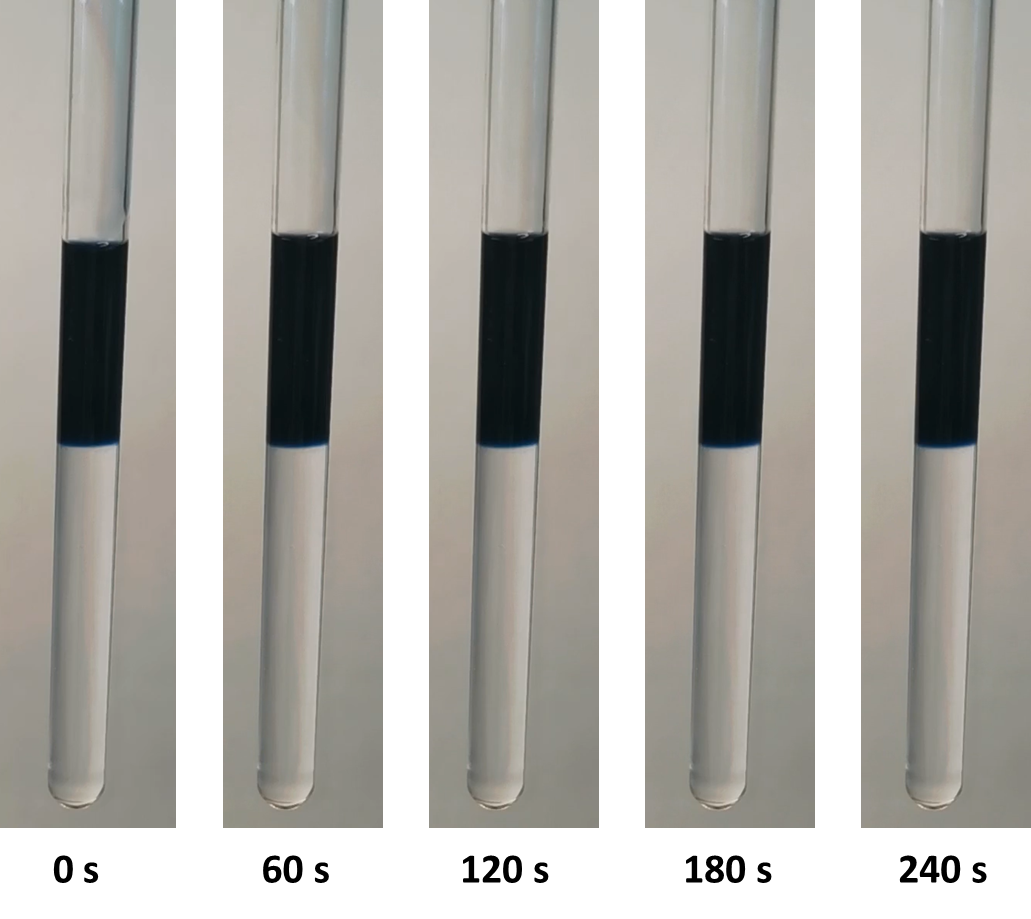


**Fig. S5.** Photographs of the addition of acetyl chloride with a small amount of oil red O into HemimTf_2_N.


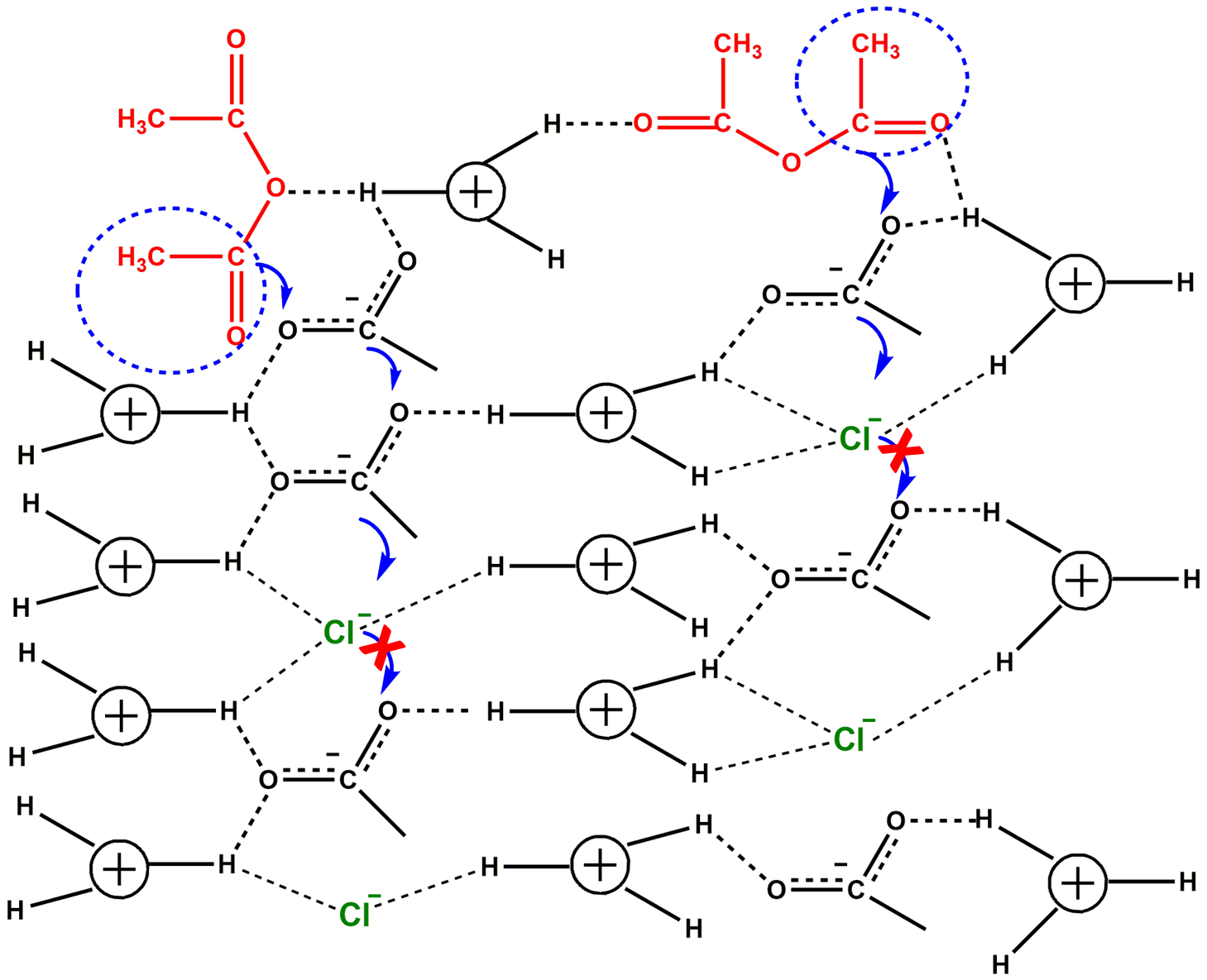


**Fig. S6.** Schematic diagram of the transmission of acetic anhydride in the mixture of RʹmimAc and RʹmimCl.

**Table S2.** Reaction conditions and results of the cellulose acetylation in the EmimAc/EmimCl mixture.


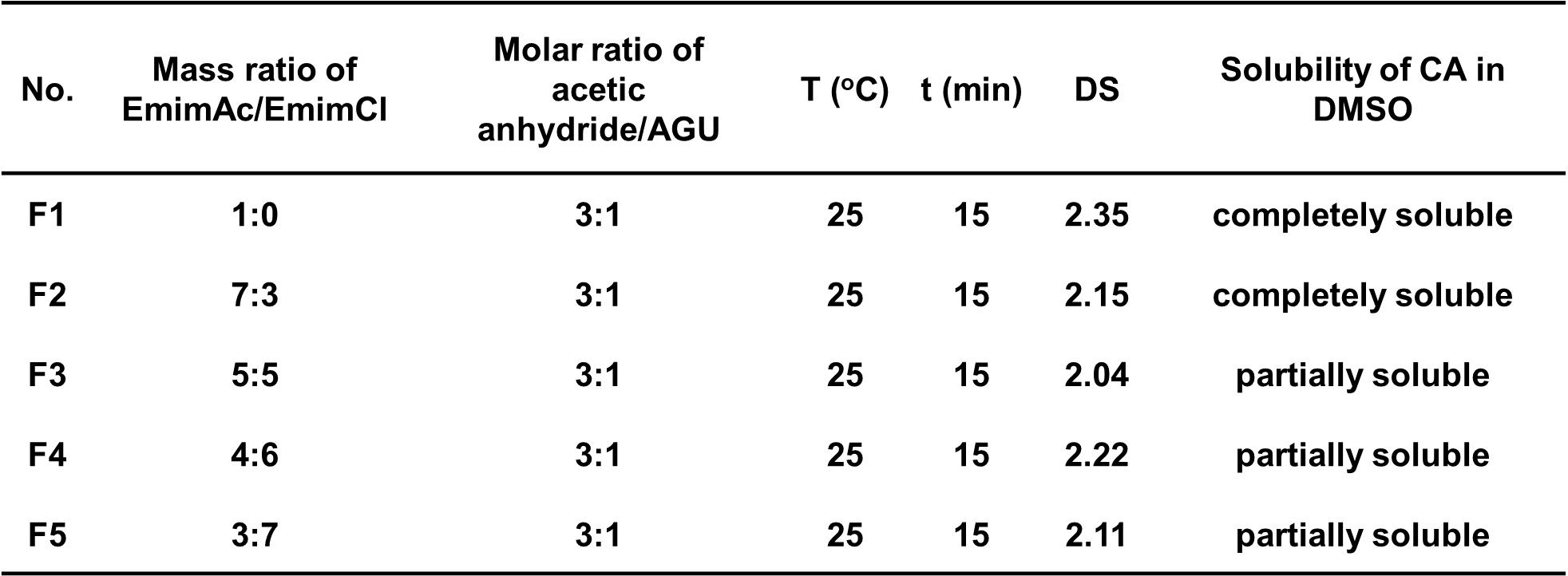


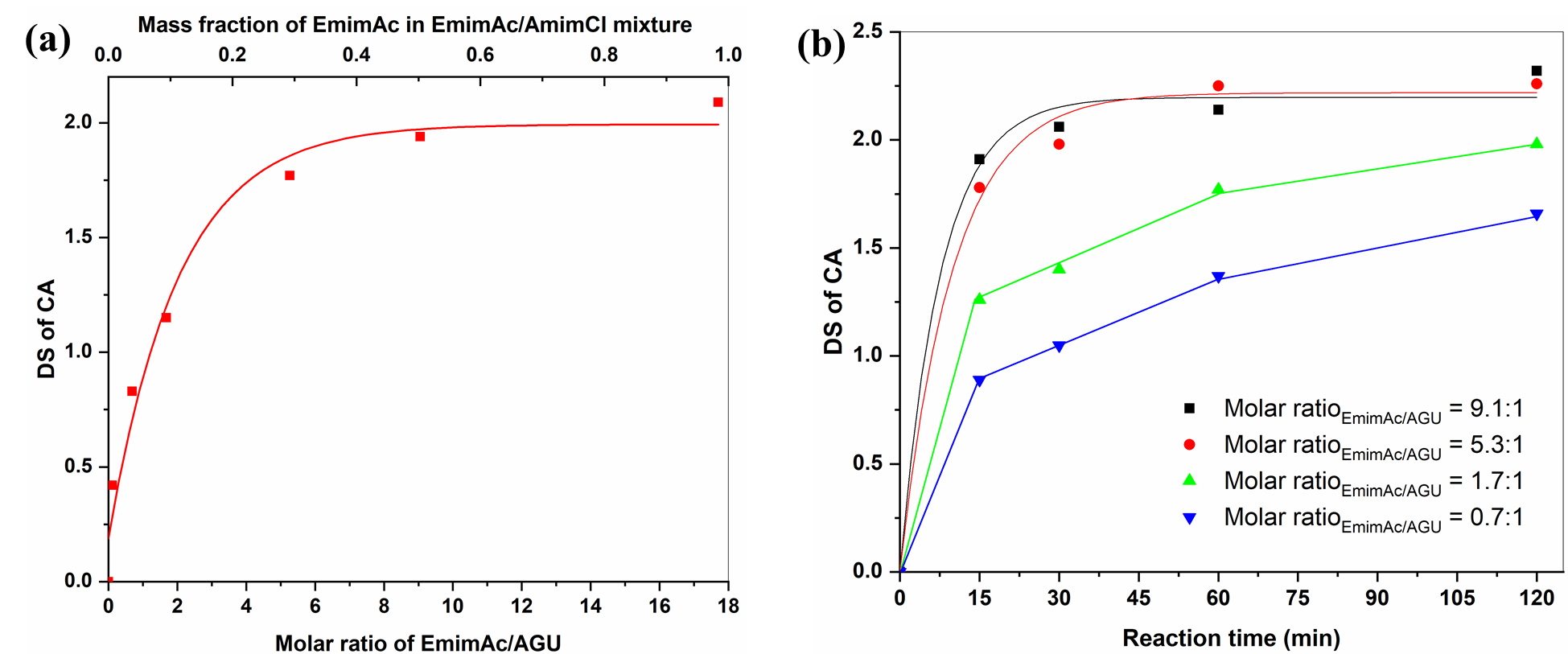


**Fig. S7.** Reaction conditions and results of the cellulose acetylation in the EmimAc/AmimCl mixture. The DS belongs to the soluble acetylated cellulose.

**Table S3.** Reaction conditions and results of the cellulose propionation in 1-butyl-3-methylimidazolium propionate ([Bmim][CH_3_CH_2_COO]).


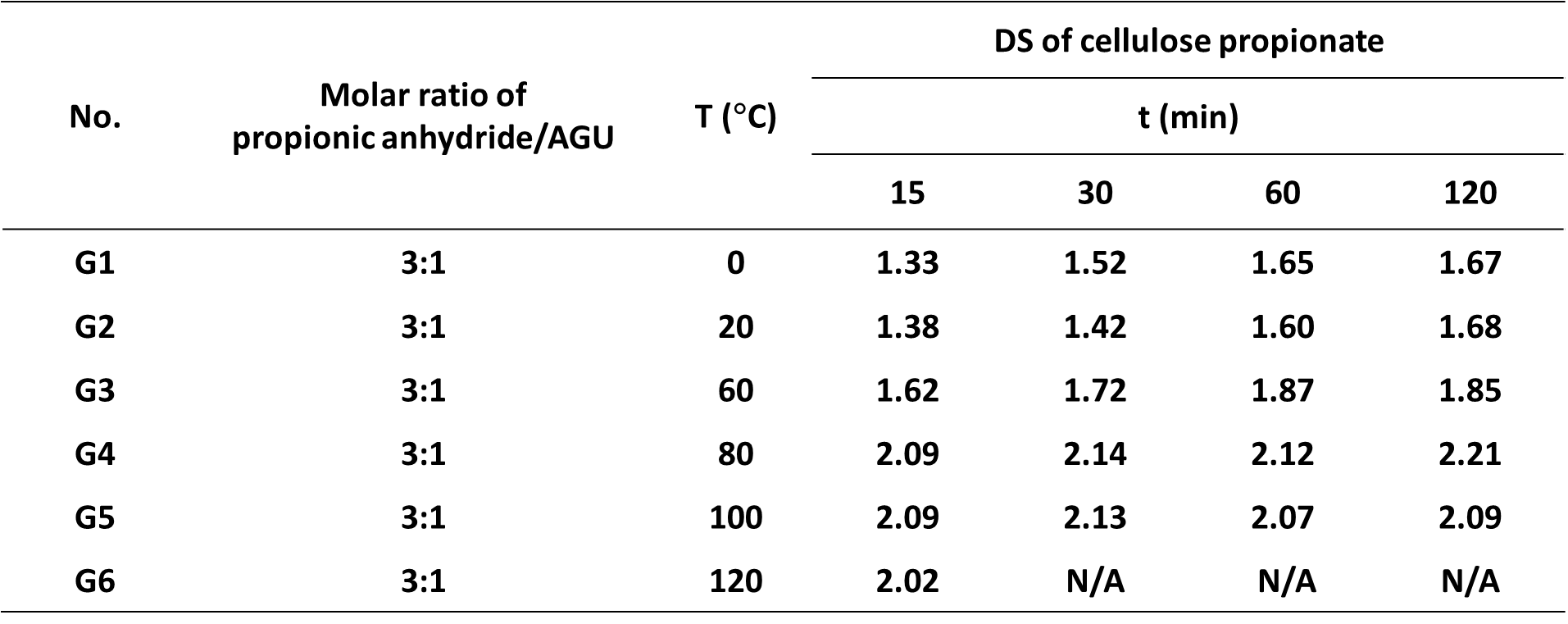


**Table S4.** Reaction conditions and results of the cellulose benzoylation in 1-butyl-3-methylimidazolium benzoate ([Bmim][PhCOO]).


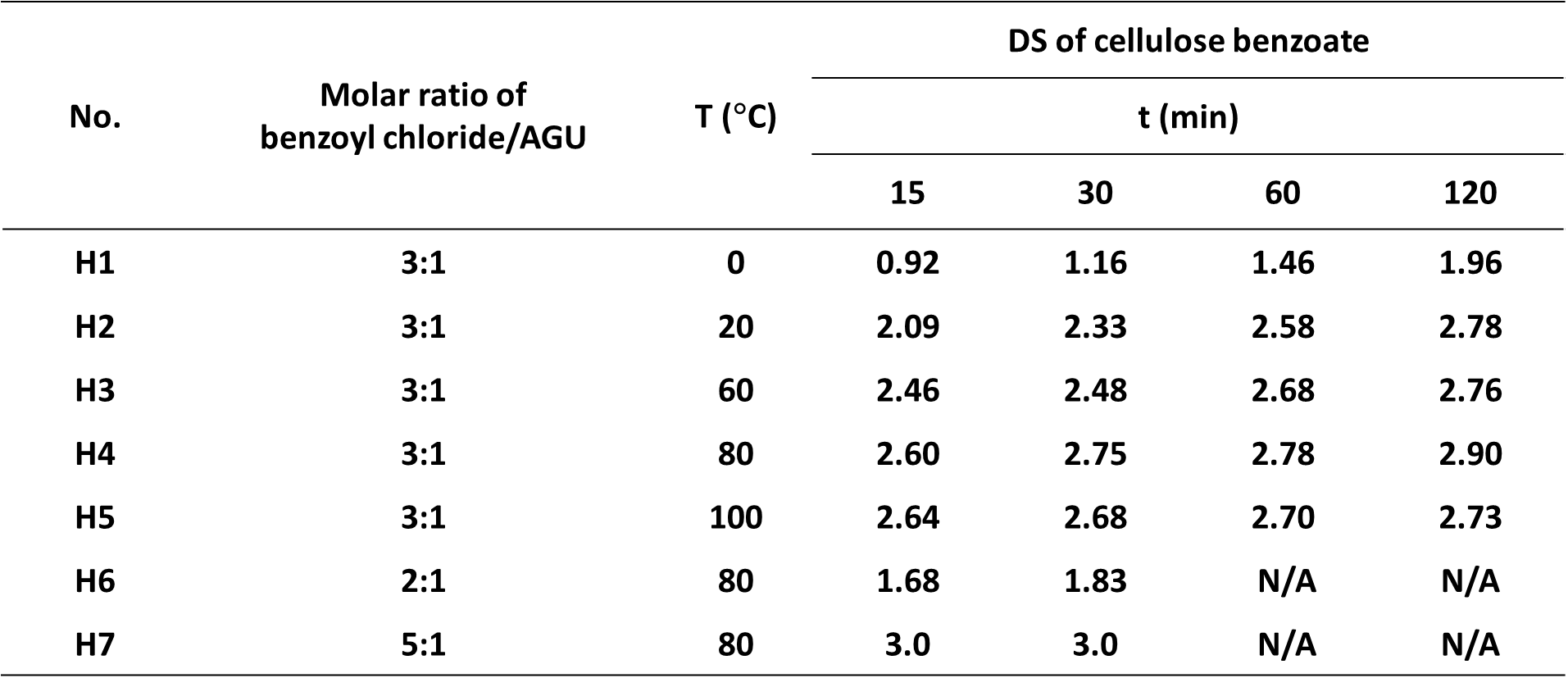


**Table S5.** Reaction conditions and results of the cellulose cyclohexyl formylation in 1-butyl-3-methylimidazolium cyclohexanecarboxylate ([Bmim][ChCOO]).


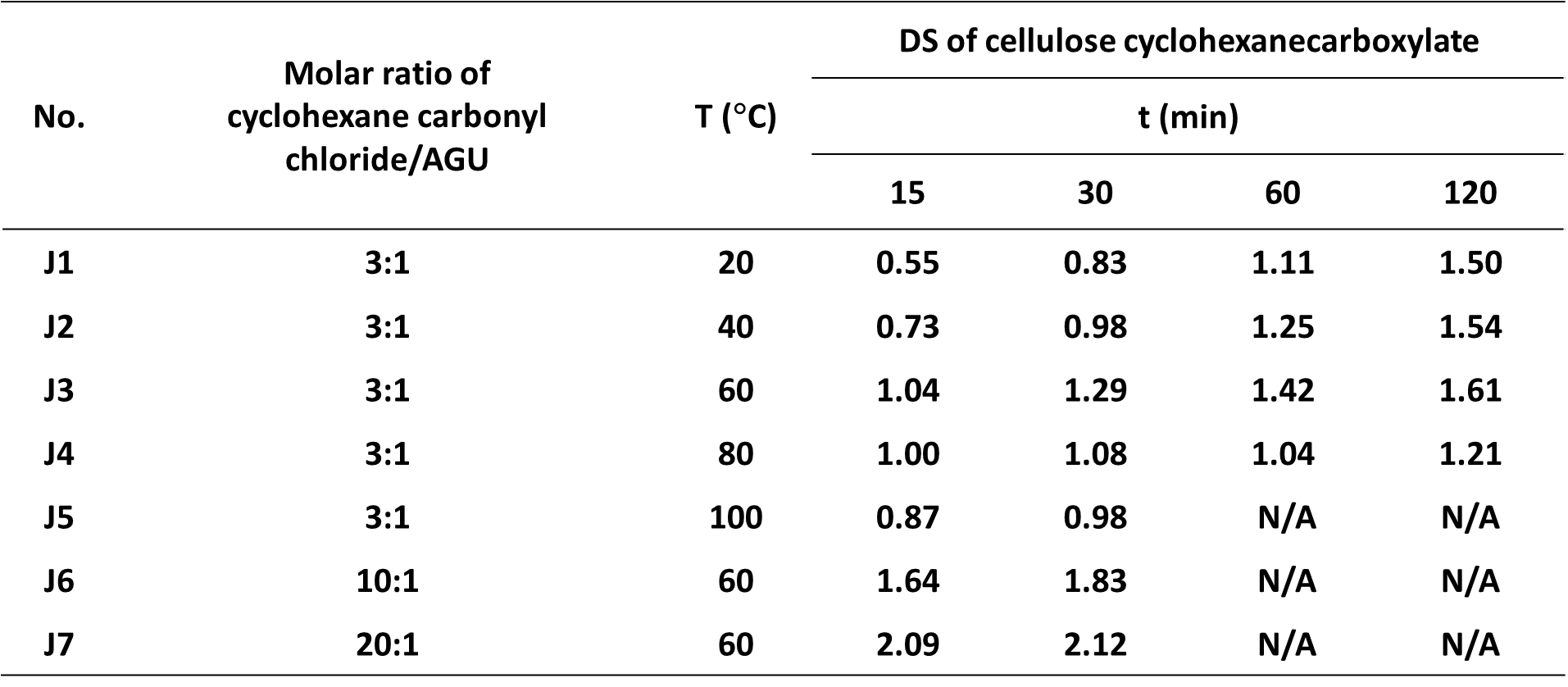


**References**

1. Zhang, H.; Wu, J.; Zhang, J.; He, J. S. 1-Allyl-3-methylimidazolium chloride room temperature ionic liquid:  a new and powerful nonderivatizing solvent for cellulose*. Macromolecule***s 2005***,* 38, 8272-8277.
